# Health gains for users of long-term home care services in Portugal

**DOI:** 10.1590/0034-7167-2024-0121

**Published:** 2025-03-10

**Authors:** Susana Alexandra Fonseca-Teixeira, Pedro Miguel dos Santos Dinis Parreira, Lisete dos Santos Mendes Mónico, João Manuel da Costa Amado

**Affiliations:** I Universidade Católica Portuguesa. Porto, Portugal; II Escola Superior de Enfermagem de Coimbra, Unidade de Investigação em Ciências da Saúde: Enfermagem. Coimbra, Portugal; III Universidade de Coimbra Faculdade de Psicologia e de Ciências da Educação. Coimbra, Portugal

**Keywords:** Home Care Services, Health Gains, Long-Term Care, Aging, Nursing, Servicios de Atención de Salud a Domicilio, Logros en Salud, Cuidados a Largo Plazo, Envejecimiento, Enfermería

## Abstract

**Objectives:**

to evaluate health gains sensitive to nursing care in the context of long-term home care.

**Methods:**

this was a quantitative, retrospective study carried out in the north of Portugal. The sample consisted of 151 users aged 18 or over. Descriptive and correlational analysis, non-parametric tests and exploratory factor analysis were carried out.

**Results:**

the results revealed that the patients admitted were an ageing, dependent population with multimorbidities and low potential for rebuilding autonomy. The care provided by the home care team had a positive impact on improving functionality, controlling symptoms, reducing pressure ulcers and the risk of falls.

**Conclusions:**

there is an urgent need for effective investment in promoting home care, guaranteeing timely and equitable access to health care. It is necessary to invest in effective, efficient public policies, driven by social and economic sustainability, in order to guarantee better health outcomes for the population.

## RESUMEN

**Objetivos:**

evaluar los beneficios para la salud sensibles a los cuidados de enfermería en el contexto de la atención domiciliaria continuada.

**Métodos:**

es un estudio cuantitativo y retrospectivo realizado en la región norte de Portugal. La muestra estuvo compuesta por 151 usuarios mayores de 18 años. Se realizaron análisis descriptivos y correlacionales, pruebas no paramétricas y análisis factorial exploratorio.

**Resultados:**

los resultados demostraron que los pacientes ingresados son una población envejecida, dependiente, con multimorbilidad y escaso potencial para recuperar su autonomía. Los cuidados prestados por el equipo de atención domiciliaria repercutieron positivamente en la mejora de la funcionalidad, el control de los síntomas, la reducción de las úlceras por presión y el riesgo de caídas.

**Conclusiones:**

es menester invertir de forma efectiva en la promoción del cuidado domiciliario, garantizando el acceso oportuno y equitativo a la atención sanitaria e invertir en políticas públicas eficaces y eficientes, impulsadas por la sostenibilidad social y económica que garantice mejores resultados para la población.

## INTRODUCTION

Demographic ageing has increased exponentially in recent decades, and this has led to new challenges for health systems and services, resulting from the increase in the chronicity of socio-health problems and the growing prevalence of functional dependence, with important implications and direct impacts on all areas of society^(1)^. This situation has led to an increasing demand for health care and, consequently, the need for nursing care, with repercussions on economic and social sustainability. The situation has led several countries to rethink new policy strategies in order to respond cost-effectively to the increased need for care and to guarantee the future sustainability of health services through a more appropriate and effective policy response that meets the needs of the population^(2,3)^.

In this sense, European Union policies have stressed the importance of all states having access to adequate, quality and affordable long-term care services, provided by qualified professionals, in order to encourage deinstitutionalization and prioritize home care and community services through investment in preventive and rehabilitation care^(4,5)^.

Against this backdrop of rapid population aging, many countries are investing in home-based long-term care as a response that has favorable strategic potential for services, users and the community^(6)^. In Portugal, too, the National Network for Integrated Continuing Care was created in 2006 as a response to new social and health needs^(7)^, and it includes the Integrated Continuing Care Teams (ICCT), as a home support response and a closer solution, which allow care to be provided by enhancing integrated services in the community^(6)^. ICCTs make it possible to set up a set of home healthcare responses with a major impact in terms of population coverage and guarantee adequate accessibility to healthcare^(8,9)^.

Among European countries, Portugal is experiencing a marked ageing process, opening up space for a sustained increase in demand for integrated long-term care.

Based on these issues, this study set out to evaluate the health gains associated with nursing care for dependent people at home, with a view to continuously improving quality and contributing to the definition of prevention strategies that are consistent with achieving health gains and supporting decision-making.

## OBJECTIVES

To evaluate health gains sensitive to nursing care in the context of ICCT.

## METHODS

### Ethical aspects

Considering the ethical aspects of scientific research, this study was submitted to the Ethics Committee for Health of the Northern Regional Health Administration, obtaining approval in 2020, whose opinion is attached to this submission, in accordance with the Declaration of Helsinki^(10)^.

During the collection and statistical processing of the data, the anonymization of the data collected was respected and guaranteed, by assigning an identification code to each user, in order to de-identify the collection and the possibilities of identification by third parties. Only quantifiable and non-identifiable data was used to build the database.

### Study design, period and location

A retrospective quantitative methodology was used and a correlational and predictive study was carried out, guided by the SQUIRE tool. The study was carried out in an ICCT in the northern region of Portugal, covering the period from 2017 to 2020.

### Population; inclusion and exclusion criteria

The population consisted of all the users (n = 151) receiving home care at an ICCT in the north of Portugal.

The inclusion criteria were: patients admitted to the ICCT during the defined time period and aged 18 or over. The exclusion criterion was being under the age of 18. In this way, 151 users aged 18 or over were assessed over this period of time.

### Study protocol

In this study, we consulted and collected data from the clinical files in the SClínico information system - nursing profile and in the Information System of the National Network for Integrated Continuing Care. Data was collected at the time of referral, which in this study is referred to as T0; at admission, referred to as T1; and at discharge, referred to as T2, using a data collection grid designed by the researcher.

This study used the Nursing Care Effectiveness Model, which is anchored in Donabedian’s Health Quality Model and includes structure, process and results^(11,12)^.

The structure variables refer to the sociodemographic characteristics of the users (age, gender, marital status, education, disease history).

The process variables indicate the nursing interventions in the areas of focus: self-care; functional status; cognitive status; symptoms (pain); and the nursing diagnoses (risk of constipation, constipation, nausea, risk of ankylosis, ankylosis, walking, walking aid, management of the therapeutic regime; role of the caregiver, stress on the caregiver, muscle movement and body balance).

The outcome variables refer to the users’ functional status because it emerges in the literature as a measure that covers the way people carry out their Activities of Daily Living (ADLs) and because it allows for a positive perspective on the results. In addition, functional status, as an outcome measure, has the potential to be sensitive to nursing care, since a large part of nurses’ practice is related to diagnosing and intervening in human responses to illness and treatment^(13)^. The following instruments were used for the assessment: the Barthel scale; the Braden scale; the Morse scale; the numerical pain scale; the National Functionality Table; and the Older Adult Functionality Table. To assess the users’ individual functionality profile, we used the Elderly Nursing Core Set instrument, validated for the Portuguese population. This instrument consists of 25 items based on the International Classification of Functioning, Disability and Health, which are grouped into four dimensions: Self-care; Learning and mental functions; Communication and relationships with friends and caregivers. Each item is evaluated according to a five-point Likert scale, which classifies the functional profile. Thus, the total score is obtained from the sum of all the items, ranging from 0 to 125 points, and the higher the score, the worse the patient’s functional profile^(11,14,15,16)^.

Given that some of the scales had different metrics, in order to be able to combine the different databases, we standardized the measurement scales and reconverted them according to a four-point Likert scale corresponding to the level of functionality: (1) no disability [0-4%]; (2) mild to moderate disability [5-49%]; (3) severe disability [50-95%]; and (4) complete disability [96-100%]. We carried out the evaluation and factor analysis of the main components and obtained four constructs: Mobility (walking at home/inside buildings; walking on the street; walking on stairs); ADL (using the toilet, potty and/or urinal; lying down/getting out of bed; sitting down/getting up from chairs; dressing/undressing; washing/bathing; controlling stools; controlling urine; feeding/eating); Instrumental Activities of Daily Living (IADL) (preparing meals; doing laundry; household chores; shopping; using transportation; managing money; taking medication; using the telephone) and cognitive status (orientation in time and space).

The users’ health gains were assessed through the improvement seen in the parameters contained in the measuring instruments from the time of referral to the time of admission and discharge.

To analyze the data, we used the Statistical Package for the Social Sciences (SPSS), version 23.0. Factor analysis of the main components was carried out with Varimax rotation, sensitivity analysis using the Kolmogorov-Smirnov test, reliability analysis using Cronbach’s Alpha and repeated measures tests^(17)^.

After analyzing the asymmetry and kurtosis measures, we conducted parametric tests due to the size of the sample in each analysis group. We used correlational statistics, non-parametric tests and exploratory factor analysis.

## RESULTS

The context of this research made it possible to assess the impact of nursing care on the health gains of patients admitted to an ICCT.

The population consisted of 151 patients admitted to the ICCT, with an average age of 78.3 years (SD=13.2 years), ranging from a minimum of 22 to a maximum of 104 years. The majority (72.2%) were aged 75 or over, female (60.9%), mostly widows (47.5%). It should also be noted that 27.6% of the patients had not been to school and 9.3% (n=14) lived alone, i.e. without a caregiver.

The majority of users (64.9%) were referred by Primary Health Care functional units and 21.9% by the hospital, with the main reasons for referral being the treatment of pressure ulcers (39.7%) and rehabilitation (31.1%). The patients admitted to the ICCT had various chronic diseases and multimorbidities, the most frequent being pressure ulcers (45.6%), thrombosis/stroke (9.9%), fractures (9.3%), neoplasms (7.2%), diabetes (6.6%) and hypertension (6.6%).

Analysis of the data shows that the average delay for admission to the ICCT was 18.2 days (SD=20), with a median of 11 days, with a minimum of 2 and a maximum of 142 days.

With regard to the dependency assessment, Table 1 shows that after the ICCT intervention, users showed a significant improvement from admission to discharge in all dimensions: 29% (ηp2 = 0.29) in mobility (walking); 45% (ηp2 = 0.45) in ADL; 31% (ηp2 = 0.31) in IADL; and 28% (ηp2 = 0.28) in cognitive status.

**Table 1:** Evolution in the dimensions of functionality at the three moments (referral, admission and discharge): repeated measures and linear contrasts

Dimension	T0	T1	T2	Λ Wilks	F	η_p_^2^	Power (1-β)	Linear Contrasts		
	M	M	M					T1 *vs.* T0	T2 *vs.* T0	T2 *vs.* T1
Mobility	3.46	3.51	3.03	0.71	30.68***	0.29	1.0	0.53*	-0.42*	-0.48*
ADL	3.17	3.31	2.72	0.55	61.14***	0.45	1.0	0.15*	-0.44*	-0.6*
IADL	3.65	3.71	3.33	0.69	32.89***	0.31	1.0	0.06*	-0.32*	-0.38*
Cognitive state	2.56	2.64	2.29	0.72	28.53***	0.28	1.0	0.07*	-0.27*	-0.34*

Statistical significance: *p < 0.05 **p < 0.01 ***p < 0.001; Older adult Nursing Core Set: [1 (no disability) - 4 (complete disability (96-100%)]; M - Mean; F - F-ratio; ηp2 - Partial eta-squared; T0 - Referral to ICCT; T1 - Admission to ICCT; T2 - Discharge from ICCT; vs. – versus.

With regard to the risk of developing pressure ulcers, there was a significant overall effect, reflecting a decrease in the risk of developing pressure ulcers from admission to discharge in all dimensions: 7% (ηp2 = 0.07) in sensory perception; 5% (ηp2 = 0.05) in activities; 31% (ηp2 = 0.31) in nutrition; 14% (ηp2 = 0.14) in humidity; 9% (ηp2 = 0.09) in mobility and; 9% (ηp2 = 0.09) in friction and sliding forces. We found that there was a significant overall effect of around 35% (ηp2 = 0.35), reflecting an improvement in pressure ulcers from admission to discharge.

Analysis of the risk of falling, assessed using the Morse scale (see Table 2), showed an overall positive effect of around 3% (ηp2 = 0.03).

**Table 2:** Morse scale in referral, admission and discharge: repeated measures and linear contrasts

Scale	Reference values	T0	T1	T2	Λ Wilks	F	η_p_^2^	Power (1-β)	Linear Contrasts		
		M	M	M					T1 vs. T0	T2 vs. T0	T2 vs. T1
SCORE (Total)	0 – 125	47.58	48.64	45.36	0.97	2.57	0.03	0.51	1.06	-2.22*	-3.28*

Statistical significance: * p < 0.05; M - Mean; F - F-ratio; ηp2 - Partial eta-squared; T0 - Referral to ICCT; T1 - Admission to ICCT; T2 - Discharge from ICCT.

With regard to pain assessment, there was a significant overall effect of around 58% (ηp2 = 0.58) from admission to discharge.

Table 3 shows that patients admitted to the ICCT had a significant improvement in nursing diagnoses: 50% in the risk of constipation (χ^2^ = 11.25; p = 0.001); 39.4% in constipation (χ^2^ = 19.6; p < 0.001); 20% in nausea (χ^2^ = 8.07; p = 0.005); 68.7% in walking (χ^2^ = 17.85; p < 0.001); 54.8% in walking with a walking aid (χ^2^ = 9.98; p = 0.002); 41.7% in the management of the therapeutic regime (χ^2^ = 25.66; p < 0.001); 54% in the role of the caregiver (χ^2^ = 19.51; p < 0.001) and 44.5% in the stress of the caregiver (χ^2^ = 5.14; p = 0.023).

**Table 3:** Nursing Diagnoses at Admission and Discharge: chi-square test

Diagnósticos	T1				T2				χ2 Admission vs. Discharge
	Not compromised		Compromised		Not compromised		Compromised		
	n	%	n	%	n	%	n	%	
Risk of constipation	5	8.3	55	91.7	35	58.3	25	41.7	11.25***
Constipation	37	52.1	34	47.9	65	91.5	6	8.5	19.6**
Nausea	42	76.4	13	23.6	53	96.4	2	3.6	8.07*
Walking	1	2.1	47	97.9	34	70.8	14	29.2	17.85**
Walking aid	4	9.5	38	90.5	27	64.3	15	35.7	9.98**
Therapeutic regimen management	32	21.2	109	72.2	98	69.5	43	30.5	28.66**
Role of the caregiver	13	17.6	61	82.4	53	71.6	21	28.4	19.51***
Caregiver stress	7	25.9	20	74.1	19	70.4	8	29.6	5.14*

Statistical significance: *p < 0.05 **p < 0.01 ***p < 0.001; n - Sample size; % - percentage; X^2^ - Chi-square test statistic; T1 - Admission to ICCT; T2 - Discharge from ICCT.

Table 4 also shows that, after the nursing intervention by the ICCT team, the patients showed an improvement in muscle movement impairment from admission to discharge, showing gains in movement (Mean rank_muscular_movement T2 = 63.68; Mean rank_muscular_movement T1 = 93.32; Z = -4.28; p < 0.001). With regard to body balance, there was also an improvement in body balance impairment from admission to discharge, so that at the time of discharge, balance impairment was lower than at admission (Mean Rank_body_balance T2 = 61.56; Mean rank_body_balance T1 = 83.44; Z = -3.27; p < 0.001), indicating gains in body balance. The results seem to indicate that the nursing interventions have a positive effect.

**Table 4:** Mean, mean rank and significance of nursing diagnoses (muscle movement and body balance)

Diagnostics	T1			T2			Teste Mann-Whitney			
	M	Ranks		M	Ranks					
		Mean rank	Sun of ranks		Mean rank	Sun of ranks	U	W	Z	p
Muscular Movement	3.12	93.32	7279.0	2.42	63.68	4967.0	1886.0	4967.0	-4.28	0.000
Body Balance	2.67	83.44	6008.0	2.07	61.56	4432.0	1804.0	4432.0	-3.27	0.001

*Scale: [1 (no impairment) - 4 (highly impaired); M - Mean; U - Mann-Whitney test statistic; W - Wilcoxon test; Z - Data point in standard deviation units; p - Statistical significance; T1 - Admission to ICCT; T2 - Discharge from ICCT.*

We would point out that the results of this investigation showed that there was a significant deterioration in the health status of users during the time between referral and admission. This analysis shows that, while waiting to be admitted to the ICCT, users suffer a significant deterioration (p < 0.05) in terms of mobility (walking), ADLs, IADLs and cognitive status (see Table 1). Regarding the risk of developing a pressure ulcer, from referral to admission, there was a significant worsening (p < 0.05) in 3 of the dimensions: nutrition, humidity and friction and sliding forces. There was a significant increase (p < 0.05) in pain from the time of referral to admission.

It should be noted that users who were monitored by the ICCT showed health gains that were sensitive to nursing care. In this way, we can emphasize that this study showed that there was a worsening of the health condition of users during the waiting time for admission and an improvement during hospitalization in the ICCT.

## DISCUSSION

The aim of this study was to evaluate the health gains that are sensitive to nursing care in the context of ICCT.

With regard to the sociodemographic profile, it was possible to identify that the users admitted to the ICCT are mostly elderly, with multimorbidity, low levels of literacy and high levels of dependence on mobility (walking), self-care for ADLs, IADLs and orientation in relation to time and space, indicating low potential for rebuilding autonomy and, therefore, great vulnerability in their health condition.

This study showed that there were unequivocal gains in health, as a result of the evaluative metrics, supported by the statistical tests of repeated measures, which showed that the care provided by the multi-professional team translated into health gains in terms of mobility (walking), ADLs, IADLs, cognition, risk of developing a pressure ulcer, risk of falling and a reduction in the number and severity of pressure ulcers and pain. Also noteworthy was the significant improvement in the nursing diagnoses of patients admitted to the ICCT as a result of the tests carried out.

This study also showed that the waiting time between the time of referral and admission to the ICCT worsened users’ health conditions in terms of mobility (walking), self-care for ADLs and IADLs, orientation in time and space, risk of falls and risk of pressure ulcers.

However, despite ICCT still being one of the areas of healthcare with the least investment, this research showed health gains in a disadvantaged referral sample, with multimorbidity and low levels of literacy.

Also, the length of referral times calls for an urgent need to speed up the processes of referring users, which will help to reduce the average length of stay of users admitted to hospitals, with the resulting socio-sanitary and economic benefits. One of the consequences of the fragmentation of care is the slowness of referrals, and this problem is accentuated during hospital discharges. Studies show that a 10% increase in home care is associated with a 1.2% to 2.1% reduction in hospital stays, contributing to a reduction in the high costs to the National Health System^(18)^.

Therefore, there is an urgent need, from a political point of view, for a clear position and a commitment to ICCT. It is therefore important to integrate care, as we realize that delay not only worsens health indicators, as shown in this study, but also reduces healthcare costs and improves users’ quality of life^(8,19)^.

In addition, studies show the importance of encouraging the creation of more home care teams and equipping existing teams with appropriate and specialized professionals, with the allocation of adequate time and resources for specific and effective intervention in the provision of care to users^(6,9)^.

There is an urgent need for good integration and interconnection of care with all systems and to invest in public policies in order to guarantee better health outcomes for the population. Although health policies only show results in the medium and long term, in order for them to become sustainable, there needs to be continuity in investing in effective, efficient, inclusive and innovative solutions, defined by values and evidence, driven by social, economic and environmental sustainability^(20,21)^. Faced with the complexity of care, especially with the increase in dependent users whose trend is worsening, we need to rethink the logic of integration and, above all, continuity of care with multidisciplinary, structured and detailed care plans throughout the user’s health journey. A new budgetary logic of harmonizing public policies is therefore essential, based on well-being objectives and overcoming the current fragmentation, lack of coordination and discontinuity of health care, by integrating and managing people’s journeys through the health services they need.

In this way, assessing the impact of nursing interventions on the health gains of users receiving nursing care within the scope of ICCT has become essential, with a view to improving health care.

### Study limitations

Although the study has contributed to increasing knowledge about the health gains of users admitted to the ICCT, a possible limitation is the fact that it was a convenience sample, carried out only in an ICCT whose reality may not be similar to that of other units and it may be imprudent to generalize the results.

### Contributions to the field of nursing, health and public policy

The results of this study are relevant to both Portuguese and international policymakers. We concluded that users admitted to the ICCT improved during their hospitalization. There is therefore a need for greater investment in the provision of home care, and a more effective commitment to health policies with the implementation of strategies to promote and develop home care.

In view of this problem, research into the efficiency and effectiveness of ICCTs is essential, with a view to improving healthcare, associated with a reduction in costs and the quality provided.

## CONCLUSIONS

The demographic changes that have taken place in recent decades, as a result of socio-economic progress and the important medical advances of the 20th century, have created new challenges posed by an ageing population with repercussions on economic and social sustainability. The increase in chronicity and disability problems implies reorganization with health planning, management of provision and allocation of resources.

In order to provide a more adequate and effective response to the needs of the population, it is important to adopt new policy strategies to respond, in terms of cost-effectiveness, to the increase in the need for care and to guarantee the sustainability of health policies. In this sense, policies must reinforce the importance of creating conditions for access to quality, affordable long-term care services provided by qualified professionals, prioritizing home care and community services through investment in preventive and rehabilitation care. It is therefore necessary to involve the participation and collaboration of the various social and health services as partners, in order to guarantee the effective continuity of the necessary care, making it global.

The results that emerged from the study show that the users monitored by the ICCT during the period studied were mostly elderly, had high levels of dependency, multimorbidities and low potential for rebuilding autonomy. There was an improvement in the users’ health from the moment of admission to discharge in all the parameters assessed, thus achieving health gains with the ICCT intervention. We would also point out that the users’ health condition was worsened during the waiting time until admission to the ICCT.

The results of this research have shown that there is an urgent need for more effective health policies in Portugal, with the implementation of strategies to promote and develop home-based care that is tailored to the resident pyramid structure, particularly the population and age groups most at risk. Overall, it is essential to guarantee a more resilient, inclusive, efficient and sustainable National Health Service for everyone, as a way of ensuring that users have access to the healthcare they really need.

It will therefore be essential to strengthen Primary Health Care in its mission, given that it is the gateway for users and the pillar of the National Health Service.

It is necessary to protect the most disadvantaged in society and, therefore, effectively implement the intersectoral integration of the different levels of care, between health and social care providers, through facilitating instruments that promote and facilitate user access to health care.

It is necessary to organize health services and move from referral to the integration of care centered on people and their health paths, so that users receive continuous and ongoing care throughout the different paths of life. In this way, ICCTs are an essential response and a closer solution that allows monitoring and training of users and caregivers.
