# Supplementary material for: Health gains for users of long-term home care services in Portugal
Source: Rev Bras Enferm. 2025 Mar 10;78(1):e20240121. doi: 10.1590/0034-7167-2024-0121 (PMC11893055; doi:10.1590/0034-7167-2024-0121)
Supplement: Translation

# Translation: Ganhos em saúde dos utentes com serviços de assistência domiciliar de longa duração em Portugal

**DOI:** 10.1590/0034-7167-2024-0121pt

**Authors:** Susana Alexandra Fonseca-Teixeira, Pedro Miguel dos Santos Dinis Parreira, Lisete dos Santos Mendes Mónico, João Manuel da Costa Amado

**Affiliations:** I Universidade Católica Portuguesa. Porto, Portugal; II Escola Superior de Enfermagem de Coimbra, Unidade de Inveetigação em Ciências da Saúde: Enfermagem. Coimbra, Portugal; III Universidade de Coimbra Faculdade de Psicologia e de Ciências da Educação. Coimbra, Portugal

**Keywords:** Serviços de Assistência Domiciliar, Ganhos em Saúde, Assistência de Longa Duração, Envelhecimento, Enfermagem

## Abstract

**Objetivos:**

avaliar os ganhos em saúde sensíveis aos cuidados de enfermagem em contexto dos cuidados continuados domiciliários.

**Métodos:**

estudo quantitativo, retrospectivo, realizado na região norte de Portugal. A amostra foi constituída por 151 utentes com idade igual ou superior a 18 anos. Realizou-se a análise descritiva, correlacional, testes não paramétricos e análise fatorial exploratória.

**Resultados:**

os resultados revelaram que os utentes admitidos são uma população envelhecida, dependente, com multimorbilidades e baixo potencial de reconstrução de autonomia. Os cuidados prestados pela equipe de cuidados continuados domiciliários tiveram um impacto positivo na melhoria da funcionalidade, controle de sintomas, redução das úlceras por pressão e risco de quedas.

**Conclusões:**

urge uma aposta efetiva na promoção dos cuidados domiciliários, garantindo o acesso atempado e equitativo aos cuidados de saúde. É necessário investir em políticas públicas eficazes, eficientes, impulsionadas pela sustentabilidade social e económica de forma a garantir melhores resultados de saúde para as populações.

## INTRODUÇÃO

O envelhecimento demográfico teve um aumento exponencial nas últimas décadas pelo que fez emergir novos desafios aos sistemas e serviços de saúde, resultantes do aumento da cronicidade dos problemas sociossanitários e da crescente prevalência da dependência funcional com implicações importantes e impactos diretos em todos os domínios da sociedade^(1)^. Tal situação condiciona uma procura crescente dos cuidados de saúde e, consequentemente, das necessidades de cuidados de enfermagem, com repercussões na sustentabilidade econômica e social. A situação tem levado vários países a repensar novas estratégias políticas a fim de responderem, em termos de custo-eficácia, ao aumento da necessidade de cuidados e de garantia futura da sustentabilidade dos serviços de saúde através de uma resposta política mais adequada e eficaz e que vá ao encontro das necessidades da população^(2,3)^.

Nesse sentido, as políticas da União Europeia sublinharam a importância de todos os Estados terem acesso a serviços de cuidados de longa duração adequados, de qualidade e acessíveis, prestados por profissionais qualificados, de forma a fomentar a desinstitucionalização e priorizar os cuidados domiciliários e serviços da comunidade através do investimento em cuidados preventivos e de reabilitação^(4,5)^.

Perante este cenário de rápido envelhecimento populacional, muitos países estão a investir em cuidados continuados domiciliários como uma resposta que possui um potencial estratégico favorável para os serviços, os utentes e a comunidade^(6)^. Também, em Portugal, em 2006, foi criada a Rede Nacional de Cuidados Continuados Integrados, como resposta às novas necessidades sociais e de saúde^(7)^ e, nela enquadradas, as Equipes de Cuidados Continuados Integrados (ECCI), como resposta de apoio domiciliário e solução de maior proximidade, as quais permitem a prestação dos cuidados, através da potenciação de serviços integrados na comunidade^(6)^. As ECCI permitem configurar um conjunto de respostas de saúde no domicílio, com grande impacto em termos de cobertura populacional e garantem uma adequada acessibilidade aos cuidados de saúde^(8,9)^.

Entre os países europeus, Portugal registra um processo de envelhecimento acentuado, abrindo-se espaço para um aumento sustentado na procura de cuidados continuados integrados.

Com base nessa problemática, a presente investigação tomou, como centralidade, avaliar os ganhos em saúde sensíveis aos cuidados de enfermagem em contexto domiciliário a pessoas dependentes, no sentido de melhorar continuamente a qualidade e contribuir para a definição de estratégias de prevenção condizentes à obtenção de ganhos em saúde e de suporte às decisões.

## OBJETIVOS

Avaliar os ganhos em saúde sensíveis aos cuidados de enfermagem em contexto das ECCI.

## MÉTODOS

### Aspetos éticos

Considerando os aspetos éticos da pesquisa científica, este estudo foi submetido à Comissão de Ética para a Saúde da Administração Regional de Saúde do Norte, obtendo a aprovação no ano de 2020, cujo parecer está anexo à presente submissão, em conformidade com a Declaração de Helsínquia^(10)^.

Durante a recolha e tratamento estatístico dos dados, foi respeitada e garantida a anonimização dos dados recolhidos, através da atribuição de um código de identificação a cada utente, de modo a desidentificar a colheita e as possibilidades de identificação por terceiros. Na construção da base de dados foram utilizados apenas dados quantificáveis e não identificáveis.

### Desenho, período e local do estudo

Utilizou-se uma metodologia quantitativa retrospectiva tendo sido desenvolvido um estudo correlacional e preditivo, norteado pela ferramenta SQUIRE. O estudo foi realizado numa ECCI da região Norte de Portugal, compreendendo o período temporal de 2017 a 2020.

### População; critérios de inclusão e exclusão

A população constituiu-se pela totalidade dos utentes (n = 151) alvo de cuidados domiciliários de uma ECCI da região Norte de Portugal.

Foram definidos como critérios de inclusão: os utentes admitidos na ECCI no período temporal definido e ter idade igual ou superior a 18 anos. Como critério de exclusão foi ter idade inferior a 18 anos. Desta forma, ao longo deste período temporal, foi efetuada a avaliação de 151 utentes, com idade igual ou superior a 18 anos.

### Protocolo de Estudo

Neste estudo, foi efetuada a consulta e recolha de dados dos processos clínicos no sistema de informação SClínico – perfil de enfermagem e no Sistema de Informação da Rede Nacional de Cuidados Continuados Integrados. A recolha de dados foi realizada no momento da referenciação que, neste estudo, foi designado por T0; na admissão, designado por T1; e alta, designada por T2, com recurso a uma grelha de recolha de dados da autoria da investigadora.

Neste estudo, foi utilizado o Modelo da Efetividade dos Cuidados de Enfermagem que se encontra ancorado no Modelo de Qualidade em Saúde de Donabedian contemplando a estrutura, o processo e os resultados^(11,12)^.

As variáveis de estrutura referem-se às características sociodemográficas dos utentes (idade, sexo, estado civil, escolaridade, antecedentes patológicos).

As variáveis de processo assinalam as intervenções de enfermagem nos eixos dos focos de atenção: autocuidado; estado funcional; estado cognitivo; sintomas (dor); e os diagnósticos de enfermagem (risco de obstipação, obstipação, náusea, risco de anquilose, anquilose, andar, auxiliar da marcha, gestão do regime terapêutico; papel do prestador de cuidados, stress do prestador de cuidados, movimento muscular e equilíbrio corporal).

As variáveis de resultado referem-se ao estado funcional dos utentes por este emergir na literatura como uma medida que abrange a forma como as pessoas desenvolvem as suas Atividades de Vida Diária (AVD) e por permitir uma perspectiva positiva dos resultados. Para além disso, o estado funcional, enquanto medida de resultado, possui potencial para ser sensível aos cuidados de enfermagem, uma vez que grande parte da prática dos enfermeiros se relaciona com o diagnóstico e com a intervenção sobre as respostas humanas às situações de doença e ao tratamento^(13)^. Para a avaliação, foram utilizados os instrumentos: escala de Barthel; escala de Braden; escala de Morse; escala numérica da dor; Tabela Nacional da Funcionalidade; e Tabela de Funcionalidade do Idoso. Para avaliar o perfil individual de funcionalidade dos utentes tivemos como base o instrumento Elderly Nursing Core Set, validado para a população portuguesa. Este instrumento é constituído por 25 itens baseados na Classificação Internacional da Funcionalidade, Incapacidade e Saúde que estão agrupados em quatro dimensões: Autocuidado; Aprendizagem e funções mentais; Comunicação e relacionamento com amigos e cuidadores. Cada item é avaliado de acordo com uma escala Likert de cinco pontos, que classifica o perfil funcional. Assim, o score total é obtido com a soma de todos os itens, variando de 0 a 125 pontos, sendo que quanto maior a pontuação pior o perfil funcional do utente^(11,14,15,16)^.

Dado que algumas escalas apresentavam métricas diferentes, de forma a poder conjugar as diferentes bases de dados, efetuamos a uniformização das escalas de medidas e reconversão de acordo com uma escala Likert de quatro pontos com correspondência no nível da funcionalidade: (1), sem deficiência [0-4%]; (2), incapacidade leve a moderada [5-49%]; (3), incapacidade grave [50-95%]; e (4), incapacidade completa [96-100%]. Efetuamos a avaliação e a análise fatorial dos componentes principais e obtivemos quatro construtos: Mobilidade (andar em casa/dentro de edifícios; andar na rua; andar em escadas); AVD (usar a sanita, bacio e/ou urinol; deitar-se/levantar-se da cama; sentar-se/levantar-se de cadeiras; vestir-se/despir-se; lavar-se/tomar banho; controlar as fezes; controlar a urina; alimentar-se/comer); Atividades Instrumentais de Vida Diária (AIVD) (preparar refeições; lavar roupa; tarefas de casa; fazer compras; usar transportes; gerir dinheiro; tomar medicamentos; usar telefone) e estado cognitivo (orientação no tempo e no espaço).

Os ganhos em saúde dos utentes foram avaliados através da melhoria verificada nos parâmetros constantes dos instrumentos de medida do momento da referenciação para os momentos admissão e alta.

Para análise dos dados, recorremos ao software estatístico Statistical Package for the Social Sciences (SPSS), versão 23.0. Foi efetuada a análise fatorial dos componentes principais com rotação Varimax, análise de sensibilidade por meio do teste de Kolmogorov-Smirnov, análise de confiabilidade por meio do Alpha de Cronbach e testes de medidas repetidas^(17)^.

Após análise das medidas de assimetria e curtose, conduzimos testes paramétricos, em virtude do tamanho da amostra em cada grupo de análise. Fizemos uso da estatística correlacional, testes não paramétricos e análise fatorial exploratória.

## RESULTADOS

O contexto desta investigação possibilitou avaliar o impacto dos cuidados de enfermagem nos ganhos em saúde dos utentes admitidos numa ECCI.

A população foi constituída por 151 utentes admitidos na ECCI, com idade média de 78,3 anos (DP=13,2 anos), variando entre o mínimo de 22 e o máximo de 104 anos. A maioria (72,2%) tinha idade igual ou superior a 75 anos, era do sexo feminino (60,9 %), maioritariamente viúvas (47,5%). Destaca-se, ainda, que 27,6% dos utentes não frequentaram a escola e 9,3% (n=14) residiam sozinhos, ou seja, sem cuidador.

A maioria dos utentes (64,9%) foi referenciada pelas unidades funcionais dos Cuidados de Saúde Primários e 21,9% pelo hospital, sendo os principais motivos de referenciação o tratamento de úlceras por pressão (39,7%) e a reabilitação (31,1%). Os utentes admitidos na ECCI apresentavam várias doenças crônicas e multimorbilidades, sendo as mais frequentes as úlceras por pressão (45,6%), a trombose/acidente vascular cerebral (9,9%), as fraturas (9,3%), neoplasias (7,2%), diabetes (6,6%) e a hipertensão arterial (6,6%).

Da análise dos dados sobressai que a demora média para admissão na ECCI foi de 18,2 dias (DP=20), mediana de 11 dias, com o mínimo de 2 e o máximo de 142 dias.

No que concerne à avaliação da dependência, conforme Tabela 1, verifica-se que após intervenção da ECCI os utentes apresentaram uma melhoria significativa da admissão para a alta em todas as dimensões: 29% (ηp^2^ = 0,29) na mobilidade (andar); 45% (ηp^2^ = 0,45) nas AVD; 31% (ηp^2^ = 0,31) nas AIVD; e 28% (ηp^2^ = 0,28) no estado cognitivo.

**Tabela 1:** Evolução nas dimensões da funcionalidade nos três momentos (referenciação, admissão e alta): medidas repetidas e contrastes lineares

Dimensões	T0	T1	T2	Λ Wilks	F	η_p_^2^	Poder (1-β)	Contrastes Lineares		
	M	M	M					T1 vs. T0	T2 vs. T0	T2 vs. T1
Mobilidade	3,46	3,51	3,03	0,71	30,68***	0,29	1,0	0,53*	-0,42*	-0,48*
AVD	3,17	3,31	2,72	0,55	61,14***	0,45	1,0	0,15*	-0,44*	-0,6*
AIVD	3,65	3,71	3,33	0,69	32,89***	0,31	1,0	0,06*	-0,32*	-0,38*
Estado Cognitivo	2,56	2,64	2,29	0,72	28,53***	0,28	1,0	0,07*	-0,27*	-0,34*

Significância estatística: *p < 0,05 **p < 0,01 ***p < 0,001; Elderly Nursing Core Set: [1 (sem deficiência) - 4 (incapacidade completa (96-100%)]; M – Média; F – Razão F; ηp2 - Eta-quadrado parcial; T0 – Referenciação para a ECCI; T1 – Admissão na ECCI; T2 – Alta da ECCI; vs. – versus.

Relativamente ao risco de desenvolvimento de úlceras por pressão, verifica-se um efeito global significativo, o que reflete uma diminuição do risco de desenvolver úlceras por pressão, da admissão para a alta em todas as dimensões: 7% (ηp^2^ = 0,07) na percepção sensorial; 5% (ηp^2^ = 0,05) nas atividades; 31% (ηp^2^ = 0,31) na nutrição; 14% (ηp^2^ = 0,14) na humidade; 9% (ηp^2^ = 0,09) na mobilidade e; 9% (ηp^2^ = 0,09) na fricção e forças de deslizamento. Verificamos que existiu um efeito global significativo em torno de 35% (ηp^2^ = 0,35), refletindo uma melhoria das úlceras por pressão da admissão para a alta.

Da análise do risco de queda, avaliado através da escala de Morse, (ver Tabela 2), verificou-se um efeito global positivo em torno dos 3% (ηp^2^ = 0,03).

**Tabela 2:** Escala de Morse na referenciação, admissão e alta: medidas repetidas e contrastes lineares

Escala	Valores de Referência	T0	T1	T2	Λ Wilks	F	η_p_^2^	Poder (1-β)	Contrastes Lineares		
		M	M	M					T1 vs. T0	T2 vs. T0	T2 vs. T1
SCORE (Total)	0 – 125	47,58	48,64	45,36	0,97	2,57	0,03	0,51	1,06	-2,22*	-3,28*

Significância estatística: * p < 0,05; M – Média; F – Razão F; ηp^2^ - Eta-quadrado parcial; T0 – Referenciação para a ECCI; T1 – Admissão na ECCI; T2 – Alta da ECCI.

Relativamente à avaliação da dor, registou-se um efeito global significativo em torno de 58% (ηp^2^ = 0,58) da admissão para a alta.

Da análise da Tabela 3, verificou-se que os utentes admitidos nas ECCI tiveram uma melhoria significativa nos diagnósticos de enfermagem: 50% no risco de obstipação (χ^2^ = 11,25; p = 0,001); 39,4% na obstipação (χ^2^ = 19,6; p < 0,001); 20% na náusea (χ^2^ = 8,07; p = 0,005); 68,7% no andar (χ^2^ = 17,85; p < 0,001); 54,8% no andar com auxiliar de marcha (χ^2^ = 9,98; p = 0,002); 41,7% na gestão do regime terapêutico (χ^2^ = 25,66; p < 0,001); 54% no papel do prestador de cuidados (χ^2^ = 19,51; p < 0,001) e 44,5% no stress do prestador de cuidados (χ^2^ = 5,14; p = 0,023).

**Tabela 3:** Diagnósticos de Enfermagem na Admissão e Alta: teste qui-quadrado

Diagnósticos	T1				T2				χ2 Admissão vs. Alta
	Não comprometido		Comprometido		Não comprometido		Comprometido		
	n	%	n	%	n	%	n	%	
Risco obstipação	5	8,3	55	91,7	35	58,3	25	41,7	11,25***
Obstipação	37	52,1	34	47,9	65	91,5	6	8,5	19,6**
Náusea	42	76,4	13	23,6	53	96,4	2	3,6	8,07*
Andar	1	2,1	47	97,9	34	70,8	14	29,2	17,85**
Auxiliar de marcha	4	9,5	38	90,5	27	64,3	15	35,7	9,98**
Gestão do Regime Terapêutico	32	21,2	109	72,2	98	69,5	43	30,5	28,66**
Papel do prestador de cuidados	13	17,6	61	82,4	53	71,6	21	28,4	19,51***
Stress do prestador de cuidados	7	25,9	20	74,1	19	70,4	8	29,6	5,14*

Significância estatística: *p < 0,05 **p < 0,01 ***p < 0,001; n – Tamanho da amostra; % – percentagem; χ2 – Estatística teste qui-quadrado; T1 – Admissão na ECCI; T2 – Alta da ECCI.

Pela análise da Tabela 4, constata-se, ainda, que, após a intervenção de enfermagem da equipe da ECCI, os utentes apresentaram uma melhoria do comprometimento do movimento muscular, do momento da admissão para a alta, denotando ganhos no movimento (*Mean rank*_movimento_muscular T2 = 63,68; *Mean rank*_movimento muscular T1 = 93,32; Z = -4,28; p < 0,001). Relativamente ao equilíbrio corporal, verifica-se também uma melhoria, da admissão para a alta, do comprometimento do equilíbrio corporal, de forma que, no momento da alta, o comprometimento do equilíbrio é inferior ao comprometimento na sua admissão (*Mean Rank*_equilíbrio corporal T2 = 61,56; *Mean rank*_equilíbrio corporal T1 = 83,44; Z = -3,27; *p* < 0,001), denotando ganhos no equilíbrio corporal. Os resultados parecem indicar que as intervenções de enfermagem têm um efeito positivo.

**Tabela 4:** Média, média de ordenação e significância dos diagnósticos de enfermagem (movimento muscular e equilíbrio corporal)

Diagnósticos	T1			T2			Teste Mann-Whitney			
	M	Ranks		M	Ranks					
		Mean rank	Sun of ranks		Mean rank	Sun of ranks	U	W	Z	p
Movimento Muscular	3,12	93,32	7279,0	2,42	63,68	4967,0	1886,0	4967,0	-4,28	0,000
Equilíbrio Corporal	2,67	83,44	6008,0	2,07	61,56	4432,0	1804,0	4432,0	-3,27	0,001

*Escala: [1 (sem comprometimento) – 4 (comprometido em grau elevado); M – Média; U - Estatística para o teste de Mann-Whitney; W – Teste de Wilcoxon; Z – Ponto de dados em unidades de desvio padrão; p – Significância estatística; T1 – Admissão na ECCI; T2 – Alta da ECCI.*

Salientamos que os resultados desta investigação demonstraram que existiu um agravamento significativo do estado de saúde dos utentes durante o tempo que decorreu entre a referenciação e a admissão. Desta análise sobressai que os utentes, enquanto aguardam a admissão na ECCI, sofrem um agravamento significativo (*p* < 0,05) ao nível da mobilidade (andar), AVD, AIVD e estado cognitivo (ver Tabela 1). Relativamente ao risco de desenvolver úlcera por pressão, da referenciação para a admissão, verificou-se um agravamento significativo (*p* < 0,05) em 3 das dimensões: nutrição, humidade e fricção e forças de deslizamento. Salienta-se um agravamento significativo (*p* < 0,05) da dor do momento da referenciação até a admissão.

Assinala-se que os utentes com o acompanhamento efetuado pela ECCI evidenciaram ganhos em saúde sensíveis aos cuidados de enfermagem. Desta forma, podemos salientar que este estudo evidenciou existir um agravamento da condição de saúde dos utentes durante o tempo de espera para admissão e uma melhoria durante o internamento na ECCI.

## DISCUSSÃO

Este estudo visava avaliar os ganhos em saúde sensíveis aos cuidados de enfermagem em contexto das ECCI.

Quanto ao perfil sociodemográfico, foi possível identificar que os utentes admitidos nas ECCI são maioritariamente idosos, com multimorbilidade, baixos níveis de literacia e elevados níveis de dependência na mobilidade (andar), autocuidado para as AVD, AIVD e orientação em relação ao tempo e espaço, assinalando baixo potencial de reconstrução de autonomia e, por isso, com grande vulnerabilidade na sua condição de saúde.

Este estudo demonstrou que houve ganhos inequívocos em saúde, resultado das métricas avaliativas, sustentadas nos testes estatísticos de medidas repetidas, que evidenciaram que os cuidados prestados pela equipe multiprofissional se traduziram em ganhos em saúde ao nível da mobilidade (andar), AVD, AIVD, cognição, risco de desenvolver úlcera por pressão, risco de queda e diminuição do número e da gravidade das úlceras por pressão e da dor. Salienta-se também a melhoria significativa nos diagnósticos de enfermagem dos utentes admitidos na ECCI, resultado dos testes realizados.

Este estudo também demonstrou que o tempo de espera, entre o momento da referenciação e a admissão na ECCI, fez agravar a condição de saúde dos utentes quanto à mobilidade (andar), autocuidado para as AVD e AIVD, orientação no tempo e espaço, risco de quedas, risco de úlceras por pressão.

No entanto, apesar das ECCI ainda serem uma das áreas dos cuidados de saúde com menor investimento, esta investigação evidenciou ganhos em saúde numa amostra referenciada desfavorecida, com multimorbilidade e baixos níveis de literacia.

Também, a morosidade nos tempos de referenciação apelam para a urgência na agilização dos processos de referenciação dos utentes, a qual concorrerá para a diminuição do tempo de demora média dos utentes internados nos hospitais com os benefícios sociossanitários e econômicos daí provenientes. Uma das consequências da fragmentação dos cuidados ocorre pela morosidade da referenciação, acentuando-se este problema nas altas hospitalares. Estudos demonstram que um aumento de 10% nos cuidados domiciliários está associado a uma redução de 1,2% a 2,1% no tempo de internamento nos hospitais, contribuindo para a redução dos elevados custos para o Sistema Nacional de Saúde^(18)^.

Sendo assim, é urgente, do ponto de vista político, haver um posicionamento claro e de aposta em relação às ECCI. Logo, é importante fazer uma integração dos cuidados, pois percebemos que o atraso além de piorar os indicadores de saúde, como evidenciado neste estudo, reduz os custos com a prestação dos cuidados de saúde e melhora a qualidade de vida dos utentes^(8,19)^.

Ainda, os estudos demonstram a importância de dinamizar a criação de mais equipes com intervenção domiciliária e de dotarmos as equipes existentes com profissionais adequados e especializados, com a alocação de tempo e recursos adequados para uma intervenção, específica e efetiva na prestação de cuidados aos utentes^(6,9)^.

É urgente fazer uma boa integração e interligação dos cuidados com todos os sistemas e investir em políticas públicas de forma a garantir resultados de saúde com mais qualidade para as populações. Apesar das políticas em saúde só demonstrarem resultados a médio e longo prazo, para que se tornem sustentáveis, é necessário que exista uma continuidade no investimento em soluções eficazes, eficientes, inclusivas e inovadoras, definidas por valores e evidências, impulsionadas pela sustentabilidade social, econômica e ambiental^(20,21)^. Diante da complexidade de cuidados, principalmente com o aumento dos utentes com dependência cuja tendência é de agravamento, impõe-se repensar a lógica da integração e sobretudo da continuidade de cuidados com planos de cuidados multidisciplinares, estruturados e detalhados durante o percurso de saúde dos utentes. É indispensável, assim, uma nova lógica orçamental de harmonização das políticas públicas, a partir de objetivos de bem-estar e superar a atual fragmentação, descoordenação e descontinuidade dos cuidados de saúde, por meio da integração e gestão do percurso das pessoas através dos serviços de saúde de que necessitam.

Dessa forma, avaliar o impacto das intervenções de enfermagem nos ganhos em saúde dos utentes alvo de cuidados de enfermagem no âmbito das ECCI tornou-se essencial, perspectivando a melhoria dos cuidados de saúde.

### Limitações do estudo

Ainda que o estudo tenha contributos para o aumento do conhecimento sobre os ganhos em saúde dos utentes admitidos na ECCI, entende-se como possível limitação o fato de se tratar de uma amostra de conveniência, realizada apenas numa ECCI cuja realidade apresentada poderá não ser similar à de outras unidades e poder ser imprudente a generalização dos resultados.

### Contribuições para a área da enfermagem, saúde e política pública

Os resultados deste estudo são pertinentes tanto para os decisores políticos portugueses como internacionais. Concluímos que os utentes admitidos nas ECCI tiveram uma melhoria durante o internamento. É, por isso, necessário um maior investimento na oferta de cuidados domiciliários, sendo preciso uma aposta mais efetiva em políticas de saúde com a implementação de estratégias de promoção e desenvolvimento dos cuidados domiciliários.

Face a esta problemática, a investigação em torno da eficiência e eficácia das ECCI torna-se essencial, com vistas à melhoria dos cuidados de saúde, associada a uma redução dos custos e à qualidade prestada.

## CONCLUSÕES

As alterações demográficas que ocorreram nas últimas décadas, resultantes do progresso socioeconômico e dos importantes avanços médicos do século XX, criaram novos desafios colocados pelo envelhecimento da população com repercussões na sustentabilidade econômica e social. O aumento da cronicidade e dos problemas de incapacidade implicam reorganização com planeamento em saúde, gestão da prestação com afetação de recursos.

De forma a dar uma resposta mais adequada e eficaz às necessidades da população, é importante adotar novas estratégias políticas para responderem, em termos de custo-eficácia, ao aumento da necessidade de cuidados e garantir a sustentabilidade das políticas para a saúde. Nesse sentido, as políticas devem reforçar a importância de se criar condições de acesso a serviços de cuidados de longa duração, de qualidade e acessíveis, prestados por profissionais qualificados, priorizando os cuidados domiciliários e serviços da comunidade através do investimento em cuidados preventivos e de reabilitação. É, assim, necessário envolver a participação e colaboração dos diversos serviços sociais e de saúde como parceiros, de forma a garantir a efetiva continuidade dos cuidados necessários, tornando-os globais.

Os resultados que emergiram do estudo demonstram que os utentes acompanhados pela ECCI, no período estudado eram na maioria idosos, apresentavam níveis de dependência elevados, multimorbilidades e baixo potencial de reconstrução de autonomia. Verificou-se uma melhoria da saúde dos utentes do momento da admissão para a alta em todos os parâmetros avaliados, obtendo-se, assim, ganhos em saúde com a intervenção da ECCI. Salientamos também que a condição de saúde dos utentes foi agravada durante o tempo de espera até a admissão na ECCI.

Os resultados desta investigação demonstraram que, em Portugal, urge uma aposta mais efetiva em políticas de saúde, com a implementação de estratégias de promoção e desenvolvimento dos cuidados domiciliários e dimensionados à estrutura piramidal residente, nomeadamente efetivos populacionais e grupos etários de mais elevado risco. Em termos globais, é fundamental garantir, para todos, um Serviço Nacional de Saúde mais resiliente, inclusivo, eficiente e sustentável, como forma de garantir o acesso dos utentes aos cuidados de saúde que efetivamente necessitam.

Será, pois, fundamental reforçar os Cuidados de Saúde Primários naquela que é a sua missão dado que são a porta de entrada dos utentes e o pilar do Serviço Nacional de Saúde.

É preciso proteger os mais desfavorecidos da sociedade e, portanto, implementar efetivamente a integração intersetorial dos diferentes níveis de cuidados, entre os prestadores de cuidados da área social e da saúde, através de instrumentos facilitadores que promovam e facilitem o acesso dos utentes aos cuidados de saúde.

É necessário organizar os serviços de saúde e evoluir da referenciação para a integração de cuidados centrados nas pessoas e nos seus percursos de saúde, para que os utentes recebam cuidados contínuos e continuados ao longo dos diferentes percursos de vida. Dessa forma, as ECCI assumem-se como uma resposta imprescindível e uma solução de maior proximidade que permite acompanhar e capacitar os utentes e cuidadores.
